# Centering Frontline Health Care Workers in Digital Innovation Design to Inform the Optimization of an App for Improved Male Circumcision Follow-up in South Africa: Qualitative Usability Study

**DOI:** 10.2196/44122

**Published:** 2023-03-22

**Authors:** Sarah Day, Vuyolwethu Ncube, Lactricia Maja, Beatrice Wasunna, Jacqueline Pienaar, Geoffrey Setswe, Evelyn Waweru, Caryl Feldacker

**Affiliations:** 1 Division of Social and Behavioural Sciences, School of Public Health Faculty of Health Sciences University of Cape Town Cape Town South Africa; 2 The Centre for HIV-AIDS Prevention Studies Johannesburg South Africa; 3 The Aurum Institute Johannesburg South Africa; 4 Medic Mobile Nairobi Kenya; 5 International Training and Education Center for Health University of Washington Seattle, WA United States

**Keywords:** digital health innovation, usability, health care workers perspectives, low- and middle-income countries, two-way texting, postoperative telehealth, male circumcision, South Africa, mobile phone

## Abstract

**Background:**

Voluntary medical male circumcision (VMMC) is a safe and effective HIV prevention strategy. However, adherence to recommended in-person, postoperative follow-up is inefficient for procedures with few adverse events. Two-way texting (2wT)–based follow-up appears to be a safe and an efficient alternative to scheduled clinic visits for low-risk patients who underwent VMMC. To ensure that 2wT responds to the needs of health care workers (HCWs) and patients, HCWs were closely involved in app design using a human-centered design (HCD) approach.

**Objective:**

Embedded within an ongoing randomized controlled trial of 2wT in South Africa and complementary HCD processes of 2wT app optimization, this qualitative study aimed to use key informant interviews (KIIs) to explore the thoughts, suggestions, and opinions on and perceptions of 2wT’s usability and acceptability among HCWs involved in 2wT implementation in both urban and rural South Africa.

**Methods:**

A total of 7 HCWs using 2wT in Gauteng and the North West province participated in KIIs regarding the usability and acceptability of 2wT. HCWs were asked for their opinions on 2wT as a viable, useful, appropriate, and accessible method of postoperative VMMC care. They were also asked about 2wT-related working, exploring areas where 2wT could add to or reduce their daily tasks. The KII data were explored, coded, and analyzed by 3 qualitative researchers using thematic content analysis and the ATLAS.ti (ATLAS.ti Scientific Software Development GmbH) software.

**Results:**

Most HCWs felt confident, comfortable, satisfied, and well supported using a 2wT-based follow-up as an alternative to in-person clinical reviews. They felt that 2wT was easy to use and required little technical support after initial mentoring on how to use the 2wT system. Few noted safety concerns, as men can receive clinical guidance, reassurance, and referral via 2wT. Although fewer in-person visits reduced the in-person review workload and eased clinical flow, HCWs noted the added burden of having to interact with clients via SMS text messages on evenings or weekends. HCWs reinforced the need for enhanced postoperative counseling to ensure that 2wT patients could recognize and understood how to respond to early signs of complications. HCWs suggested a rotation to spread the evening and weekend workload and ensure swift patient responses.

**Conclusions:**

In this formative qualitative study focused on HCWs, 2wT was a highly usable alternative to in-person postoperative reviews for patients who underwent VMMC in South Africa. The HCD processes likely improved the usability and acceptability of 2wT for HCWs. HCWs supported the scale-up of 2wT given the distance from the clinic to the men’s homes and the potential for reducing workload. To ensure success, providers urged sensitizing patients to the fact that 2wT augments, but does not replace, the existing after-hours and emergency care services.

## Introduction

### Background

Health care worker (HCW) shortages are common in many low- and middle-income countries (LMICs), including those in sub-Saharan Africa (SSA), and contribute to the compromised quality of health care systems and reduced access to quality patient care [1,2]. Solutions to the HCW crisis are not simple, and many center on long-term interventions such as training, raising wages, and job satisfaction [3]. In the short term, digital health innovations hold promise to create efficiencies in health care delivery and contribute to reducing the effects of chronic understaffing. Recent responses to the COVID-19 pandemic have pushed the uptake of and reliance on mobile health (mHealth) interventions further and faster [4]. However, the pace of innovation may place more value on speed over evidence. Careful consideration of the known barriers and challenges to digital health success [5] and reflection on the equity of mHealth benefits and beneficiaries [6,7] are warranted to continue to move digital health forward.

There is also broad recognition that diverse users must be engaged in the design, development, delivery, and optimization of digital health innovations. User- or human-centered design (HCD) should better amplify the perspectives of HCWs [8]. Giving HCWs a strong voice in the development of apps designed to help them overcome challenges in health care delivery may offer solutions that contribute to sustained improvements in the quality of health care in LMICs [9], including those in SSA [10,11]. In a recent review of mHealth interventions from the HCW perspective, mobile devices enabled some HCWs to overcome the difficulties in rural and geographically challenging contexts by making it possible for them to provide health care without having to travel; moreover, the review noted that they had more time with their clients because of a digital innovation [12]. The incorporation of more HCW voices from public, LMIC settings would enrich the HCD process and further improve the fit of digital health for those primarily tasked with patient care in resource-constrained settings, where these innovations are most needed.

In South Africa, there are chronic HCW shortages across all service delivery contexts [13], including critical voluntary medical male circumcision (VMMC) services that are a core component of the National Department of Health’s (NDoH) HIV prevention activities [14]. VMMC resources are scarce, potentially decreasing the quality of service delivery [15]. A recent randomized controlled trial (RCT) in Zimbabwe found that a hybrid two-way texting (2wT) approach was a safe alternative to mandatory in-person VMMC reviews with evidence of its safety, efficiency, and usability [16-19]. The 2wT approach was also shown to be acceptable and usable for HCWs and patients who underwent VMMC in the RCT and was brought to scale [18]. In South Africa, the Aurum Institute, in partnership with its affiliate, the Centre for HIV-AIDS Prevention Studies (CHAPS) and the NDoH, provides VMMC services across priority districts [20]. Together, Aurum and CHAPS provided >950,000 male circumcisions (MCs) with a low adverse event (AE) rate. Low AE rate suggests quality care provision but may also reflect AE underreporting. VMMC client safety and program inefficiencies suggest that mHealth may offer a partial solution to chronic staff shortages and pressure to achieve VMMC targets while improving the quality of care and reporting. Thus, 2wT may provide similar benefits in the context of VMMC in South Africa.

Therefore, the Aurum Institute, in partnership with the International Training and Education Center for Health at the University of Washington, the CHAPS, and its technology partner Medic, is undertaking an RCT to test whether 2wT can provide the same safety and efficiency advantages in both urban and rural VMMC settings in South Africa. The core aim of the RCT is to apply HCD principles to 2wT design, including the assessment of 2wT’s usability and acceptability from the HCW perspective, to provide guidance to inform the adoption and expansion of 2wT in the local context. A solid evidence base of the high usability and acceptability of 2wT is imperative for VMMC programs in South Africa and other contexts that consider implementing 2wT interventions to support follow-up. The RCT and cost results are not included in this paper but will be published separately.

### Goal of This Study

In this paper, we report on an embedded, formative, qualitative study aimed at exploring the usability, acceptability, and strengths of and suggestions for 2wT with HCWs involved in the RCT planning, implementation, and evaluation. *Usability* is commonly defined as the extent to that a product can be used to complete a task efficiently, effectively, and with satisfaction [21-23]. Recently, concepts such as encouragement for use, minimization of errors, learnability, and easy maintenance have been added to broaden this definition [24]. We aimed to understand the acceptability of the 2wT system among health care providers by exploring their perceptions of the usefulness of the system and the importance of the system functions. *Acceptability* has been defined as people’s affective attitudes toward a new digital health intervention, use intentions (eg, willingness to engage with the intervention), actual use (eg, frequent interaction with the intervention), and satisfaction after engaging with the intervention [25]. Acceptability is a more nuanced concept that aims to reflect HCW’s attitudes toward a technology (ease of use, tolerance, and enjoyment of use), which may influence how they engage (interact) with a system [25]. Consequently, 2wT’s acceptability was explored through HCWs’ assessment of their interactions with the system and their attitudes toward its viability as an alternative to in-person follow-up care. High usability and acceptability are critical for the expansion of the innovation across South Africa and the SSA region.

In this qualitative study, our objective was to use key informant interviews (KIIs) to explore the thoughts, suggestions, and opinions on and perceptions of 2wT’s usability and acceptability among the HCWs involved in 2wT’s implementation in both urban and rural South Africa. We expected that these insights would aid in the understanding of the best practices for replicability and inform 2wT optimization for scale-up.

## Methods

### Theory of Change: Technology Acceptance Model

**Figure 1:**
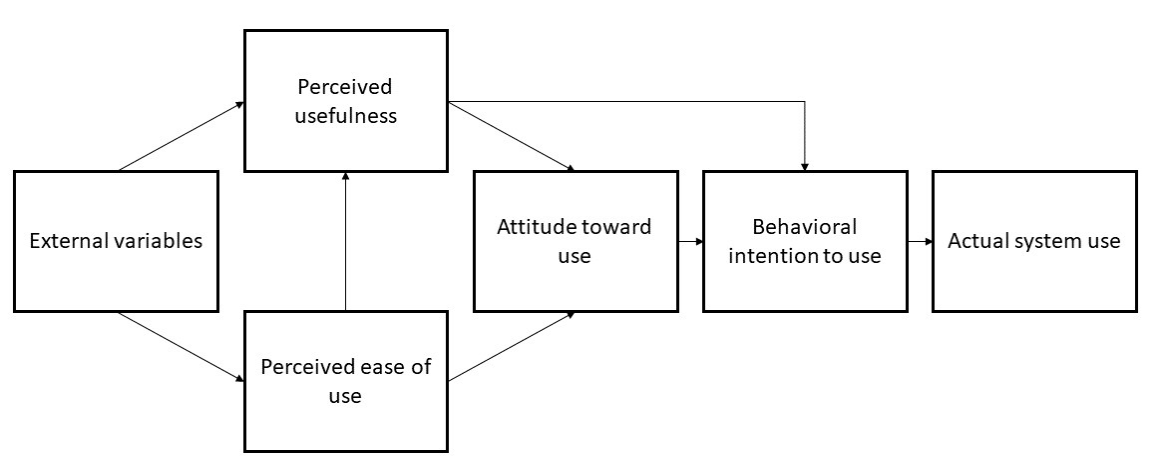
The basic technology acceptance model [34].

Web-based messaging, such as 2wT, has been proven to be effective in influencing HIV-related behaviors [26,27]. Furthermore, 2wT has been tested in other contexts in the countries in the African continent, including Zimbabwe [16-19] and Malawi [28,29]. In these contexts, HCWs found that 2wT was highly usable [17,28,29], encouraged clients to proactively engage with the healing process [17], was safe, was inexpensive, and had value [28,29]. However, the application of behavior theory can help guide the understanding of usability and acceptability to ensure optimization for each new adaptation or context. A commonly used model to understand HCWs’ and patients’ acceptance of technology is the technology acceptance model (TAM) [30]. The basic TAM presupposes that usefulness and perceived ease of use play a mediating role in the association between system characteristics (external variables) and actual system use (Figure 1). Many studies have demonstrated that the basic tenets of the TAM can be applied to determining whether a user will take up an eHealth technology [31-34]. However, most of these studies were conducted in well-resourced countries, and the applicability of the TAM in LMICs is underresearched [35].

A systematic review that examined the TAM and health informatics surmised that health care professionals are more likely to adopt new technology if they perceive that the technology can improve their performance and increase their efficacy [30]. In addition, system and information quality; clinicians’ autonomy, security, and privacy; and cultural and organizational culture were all cited as important factors that influence the uptake of new technologies within the health sector [36]. Despite the shift toward eHealth, the uptake of telemedicine in the health care sector has been poor. Therefore, HCW user rejection is an important consideration in the institutionalization of eHealth tools. In line with the systematic review by Rahimi et al [30] on studies that have drawn on the TAM model to examine HCWs’ uptake of eHealth tools, this study examined the acceptability and feasibility of 2wT, as it relates to security, confidentiality, subjective norms, facilitators, accessibility, and self-efficacy. In addition, staff’s IT experience and technical infrastructure, factors identified by Kalayou et al [35], were explored for their potential impact on eHealth technology acceptance.

### Overview of 2wT: System Design

**Figure 2:**
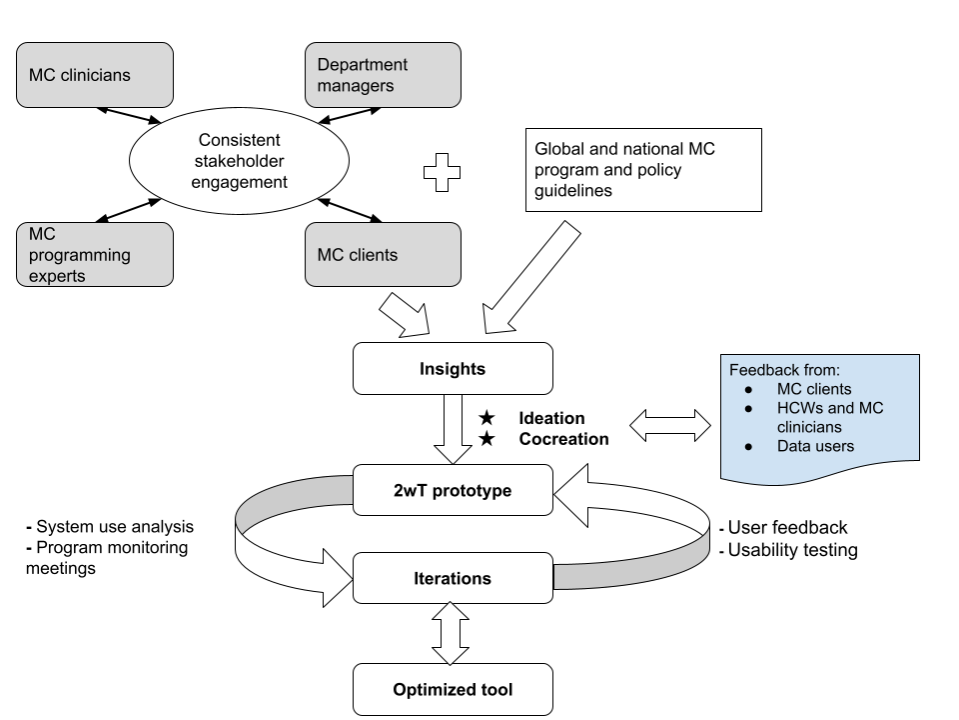
Developing and optimizing 2-way texting (2wT) for voluntary medical male circumcision (MC) postoperative care: a human-centered design process. HCW: health care worker.

The 2wT messaging platform was built using Medic’s open-source Community Health Toolkit [37]. The intervention was described in detail previously [12,16,31]. In brief, the 2wT system comprises 4 core components at the provider and patient levels: (1) hybrid automated and interactive patient-to-provider messaging over the first 13 days of post-VMMC follow-up; (2) SMS text messaging–based triaging of clients by nurses (eg, for reassurance, referrals to care, and follow-up in case of no contact); (3) daily client monitoring via SMS text messaging; and (4) longitudinal patient records (potential AEs, AE follow-up, and referral confirmation) and reporting (eg, client response rates). These features of 2wT were designed to support a streamlined workflow, reinforce high-quality VMMC services, and generate data to monitor the program delivery. The 2wT platform was designed and built using an HCD approach, which centers the user during the design development [7,38,39]. The iterative steps of HCD as related to the development of the 2wT system for VMMC reflect the following key concepts [7] can be seen in Figure 2 and Table 1 below.

**Table 1:** Iterative steps of human-centered design.

Step	Description
Discover	Working with users, including VMMC^a^ clients, clinicians, health department managers, monitoring and evaluation officers, and programming experts, to map the existing workflow Identify the key players in the system, the current channels of communication, the available infrastructure, the existing behaviors, and any inefficiencies or opportunities for impact Identify pain points and discuss makeshift work-arounds to the existing problems Gain an understanding of the broader context and ensure that interventions align with global and national VMMC program and policy guidelines
Define	System sketches to answer “how might we” questions Test assumptions of the impact logic of the intervention Assess users’ experience with, and perceptions of, different types of technologies to understand the human interactions that are implicit in the workflow
Ideate	Draft potential new system maps, including interventions and desired behaviors and outcomes Make use of the existing tools and only develop new tools when needed Focus on similar technology for accessibility
Prototype	Build low-fidelity prototypes Test ideas quickly and cost-efficiently in low-fidelity ways with users before jumping into development and implementation; prototypes can take many forms such as hand-drawn sketches and stickies, printed mock-ups, role-play scenarios, interactive InVision prototypes, and more
Test	Field test User feedback sessions to define potential iterations and refine the solution
Iterate	Continually refine and update the digital solution as needed
Learn	Observe user behavior and study impact Continual testing, validation, and use to refine and improve the solution

^a^VMMC: voluntary medical male circumcision.

The 2wT platform workflow changes were made in accordance with experiential learning, qualitative feedback in the validation phase, and participatory HCD sessions with program implementors and key stakeholders in South Africa. The 2wT platform prototypes were iteratively tested, validated, and informed by the users involved from the beginning of the development process. Continuous engagement between VMMC program experts and clinic department managers provided the initial VMMC procedures, prioritized indicators for data users, and corroborated the existing global and national policy guidelines on VMMC (Figure 2; Table 1). Engagement with site clinicians provided routine practice perspectives on the ground and an understanding of how incorporating messaging into the existing patient follow-up flows could work. Engagement with VMMC clients provided the user perspective on the use and acceptability of 2wT for postoperative care. These insights contributed to the ideation and co-design of the initial 2wT prototype, which was then deployed and tested to develop an optimized 2wT system. An example of a core improvement that resulted from this HCD process is the inclusion of a red exclamation mark in the icons for flagging potential AEs for easier task identification and prioritization by HCWs. This helps HCWs better streamline their workload, aiding efficiency. In addition, a server-side purging feature for messages older than 90 days makes it easier for HCWs to sync data. By purging data, there is more space on HCWs’ devices, which may speed up message delivery and task management.

### The 2wT Intervention

This qualitative study on 2wT’s usability and acceptability among HCWs was embedded within an RCT to determine 2wT’s safety and contribution to the reduction of HCWs’ workload. The 2wT RCT intervention itself and the clinical results from South Africa are detailed separately [40]. The 2wT tools have been discussed and visualized in other papers [18]. In brief, in lieu of the scheduled in-person visits on postoperative days 2 and 7, as is standard of care (SoC), men in the 2wT intervention received automated texts daily from days 1 to 13 and were asked to respond about their healing process via 2wT with a VMMC nurse. The 2wT VMMC nurse was provided with a tablet to interact with clients with concerns via 2wT or follow-up by phone call per their and the client’s discretion, as needed. The 2wT clients were asked to return to the clinic only for concerns or if they desired; otherwise, they healed independently at home. The 2wT system contains organizational tabs to help guide HCWs, including an alert to prompt the 2wT nurse to respond to men with potential AE concerns or to trace those who do not respond. Men in the routine arm, SoC, attended scheduled in-person VMMC postoperative review visits on days 2 and 7 after VMMC. Clients in both the 2wT and SoC arms were asked to return to the clinic on day 14 for a study-specific review to ascertain healing. The 2wT SMS text messages were free for clients to send and receive.

### Participants

The target population for the usability study was HCWs employed by the Aurum Institute and CHAPS working at VMMC static and mobile clinics within 2 districts: Ekurhuleni and Bojanala. Ekurhuleni was chosen because it is largely urban, and Bojanala was chosen because it is predominantly rural. The criteria for HCW inclusion in this study were as follows: (1) being a HCW aged ≥18 years and (2) employed in the 2wT study in Ekurhuleni, Gauteng, or Bojanala, North West province, South Africa. The participants for the research study were selected using a purposive sampling technique based on their willingness to participate in the study and the criteria for inclusion in the research study. A total of 7 HCWs participated in the research study.

### Data Collection

KIIs were conducted with 7 HCWs involved in the 2wT RCT (June 2021 to February 2022) to gauge the usability of, acceptability of, and satisfaction with 2wT; gather suggestions for intervention improvement; and identify both the facilitators of and barriers to the success of the program. The KIIs were audio recorded and transcribed. Thematic content analysis was used to analyze the data. The ATLAS.ti (version 6; ATLAS.ti Scientific Software Development GmbH) software was then used to create a spreadsheet of key themes, perceived barriers, and suggested enablers for the feasibility of the program from KIIs. Codes were compared between analysts and discussed to reach a consensus for reporting.

### Ethics Approval

This qualitative study was embedded in an RCT, “Expanding and Scaling Two-Way Texting to Reduce Unnecessary Follow-up and Improve Adverse Event Identification Among Voluntary Medical Male Circumcision (VMMC) Participants in the Republic of South Africa,” registered at ClinicalTrials.gov (NCT04327271). Ethical clearance for the RCT, including this substudy, was provided by the internal review boards of the University of Washington (study 00009703; principal investigator: CF) and the University of the Witwatersrand and the Human Research Ethics Committee (ethics reference number: 200204; principal investigator: GS). KII participants were given information sheets detailing the purpose of the research study, privacy, and confidentiality protection as part of the written consent process.

## Results

### Overview

The following main themes were identified: support for 2wT as a benefit for VMMC clients, support for 2wT as a benefit for HCWs, and challenges of 2wT. Each theme had several subthemes, as discussed below. Table 2 summarizes the themes and subthemes.

**Table 2:** Thematic analysis results.

Theme	Subthemes	Description
Support for 2wT^a^: HCWs^b^ note 2wT’s benefits for clients	The 2wT system provides high-quality, accessible health care for clients The 2wT system saves client’s cost and time	Client-level benefits of 2wT
Support for 2wT: 2wT’s benefits for HCWs	The 2wT system supports HCWs to provide quality care The 2wT system reduces HCWs’ workload and, likely, VMMC^c^ program’s cost On-site or practice-based training on 2wT is preferred	HCW-level benefits of 2wT
Challenges of 2wT	Perceived increase in the workload among short-staffed VMMC HCWs Lack of clarity on the distinction between 2wT routine and research duties Need to address 24/7 interaction requests from 2wT The 2wT system excludes some clients Technology challenges	Client-, HCW-, and system-related challenges that need consideration before 2wT scale-up

^a^2wT: two-way texting.

^b^HCW: health care worker.

^c^VMMC: voluntary medical male circumcision.

### Support for 2wT: HCWs Note 2wT’s Benefits for Clients

#### The 2wT System Provides High-Quality, Accessible Health Care for Clients

HCWs believed that 2wT offered men accessible, high-quality health care via telehealth. First, they perceived that 2wT may prevent delays in care, as men are encouraged to contact their VMMC nurse as soon as there is a problem and not wait for scheduled reviews. A 2wT nurse, voicing their perspective of a typical routine client who delayed care, said, “you see that there’s something wrong. And yet you don’t say anything, you know, because you’re waiting for a check-up” (P3). By contrast, 2wT encourages men who observe an issue with their healing to be proactive regarding their healing through daily access to a VMMC clinician via SMS text messaging.

The 2wT system provided clients with direct access to VMMC clinicians and allowed HCWs to respond to client questions and provide individualized care without requiring the clients to return to the clinic for the same support. *“*Most of them are very happy to be on the texting arm [2wT] because they know that doesn’t matter what time. They can always ask a question if something comes up. So yeah, it’s been a great help*.*” (P3). Another HCW framed 2wT’s advantage for men who are highly mobile, as 2wT is accessible at home, at work, or while traveling:

In a good way...they actually have to like, they can stay at home, do their check-ups at home, they don’t actually have to come here. Maybe, let’s say, for example, they had places to be. And then that’s how they can like, go to wherever where they wanted to go.

P6

The 2wT system also offered benefits to client care in terms of infection prevention during the COVID-19 pandemic because patients did not *“*have to come in and stand in the long queues...especially now, because 2wT really fits in this COVID thing. It just parts with all the stress of coming here to the clinic and waiting in the long queues with of a lot of people” (P6).

#### The 2wT System Saves Clients’ Cost and Time

Time and cost savings was reported by the HCWs as a primary benefit for 2wT clients. Several respondents noted similarly that 2wT “reduces...the patient’s cost for them to come here [clinic]” (P2) and “saved them a lot of time and money” (P3). The 2wT system was also perceived as convenient for clients who are employed, as it does not require them to take time off work to attend their clinic appointments:

...they are welcoming it and they took it positively. More especially the ones that are employed full time that are always at work like those who did not have money to travel or to come for check-ups on outside they find it very convenient.

P7

### Support for 2wT: 2wT’s Benefits for HCWs

#### The 2wT System Supports HCWs to Provide Quality Care

HCWs reported that the 2wT system allowed patients to reach out to clinicians quickly and efficiently. The 2wT system provided HCWs with alerts and visual cues to focus effort on men who needed support, suggesting that 2wT provides better follow-up than routine physical follow-ups. One of the benefits of 2wT is daily interaction, which helps alert clinicians to patients who may have concerns and would otherwise be seen only on days 2 and 7:

The nice thing about texting, you are in contact with your clients, you know what’s happening with them, you know what’s happening with [name]. Not everyone is not like that. Seven days, two days, they [routine men] come in there. Now you have an element of surprise. It [2wT] helps you to prevent the problem before it comes across.

P5

A 2wT clinician reinforced the advantage of consistent interaction with clients for swift AE identification and management:

I think about last year, we had that particular patient that was bleeding after he had removed the bandage, and we were able to bring him in the very next day, and he had to be reopened and cauterized. You know I think if we hadn’t caught it on 2wT, I don’t know if he would have found emergency number...And I mean, he didn’t want to come in, but he did. We were able to sort him out quite quickly before he became something more severe.

P3

#### The 2wT System Reduced HCWs’ Workload and, Likely, VMMC Program’s Cost

The 2wT approach was reported to be beneficial not only to clients but also to HCWs in terms of their workload associated with daily client reviews. In both rural and urban settings, HCWs reported that “...[2wT] reduces their work in terms of seeing the clients every day when [clients] comes to follow up check-up” (P1). In the rural context, the benefit may be greater, as it is common “to drive 100 plus KM just to see one patient” (P2). Reducing unnecessary checkups using 2wT may result in an increased number of MCs done per day, as clinicians “can focus on maybe getting new (VMMC) clients for that day to cut them instead of going to fetch those ones for follow up” (P2). This advantage may also be more acute during the peak season, when clinicians may perform >100 circumcisions per day. This benefit further translates into clients being attended to more rapidly for circumcision, thus leading to a reduction in the number of clients absconding from the queue owing to lengthy waiting times for the procedure:

Then we’ve got clients with complications that need our attention. And we also have new cases. Clients that need to be circumcised on that day. 2wT reduced the number of in-person reviews, so it makes life easy on our side as well...On that day, you find that we still having 100+ clients with those who were cut 48 hours ago who came for check-up. And then that itself puts us in a spotlight whereby we find ourselves fighting with the parents, fighting with the clients themselves and fighting with the next-of-kin, because they all need to be attended to, all of them, at the same time, which is highly impossible.

P7

HCWs identified that reduced workload would translate to reduced travel and fuel costs for the program, “which is great, because our fuel bill can be hugely expensive” (P3). Respondents also noted that reduction in unnecessary travel would reduce the pressure on the VMMC driver, typically 1 per site*.*

#### The 2wT System Is User Friendly

HCWs reported that the 2wT system is simple to use for HCWs. “The system is not complicated. It’s just it needs one to be on the system daily to get used to the things that needs to be done. It’s not complicated at all” (P1). Another HCW reported the following:

This system, I enjoyed it so much...This has been with me I didn’t have a problem with the system. It was straightforward. First basically self-explanatory. As long as they came and taught me a code it’s quite immediate. So, for me, it was not something that was like a mountain on my shoulders.

P5

#### On-Site or Practice-Based Training on 2wT Is Preferred

The need for 2wT training appeared to be reasonable, with mentoring or on-site training using the suggested method of learning. An HCW noted how once they were trained and began using 2wT for daily follow-up, they trained others in 2wT use at the facility, providing subsequent mentoring to ensure that the trainee was proficient:

Some are meant to teach someone else or train someone else with what you’re doing daily. For me, it’s easier to teach another clinician...because I’m usually doing 2wT. I will show them step by step. The following day, maybe show them again the next time then they can do it. I don’t think it’s a very difficult thing to show them. But yeah, it does need time for them to understand.

P2

### Challenges of 2wT

#### Perceived Increase in the Workload Among Short-Staffed VMMC HCWs

Although 2wT is reported to be user friendly, some HCWs, particularly those in understaffed VMMC facilities, reported that they were short staffed, so the additional work of responding to clients while performing other duties was overburdening them. *“*There’s only three clinicians and it’s a busy day. We’ve got so many clients to cut. In those particular days. It’s really hard to sit down and answer [2wT] when there are clients waiting” (P2). Several HCWs noted that 2wT only works with an effective clinical team approach where HCWs work together to balance and rotate responsibilities for performing MCs, conducting follow-up for men who want or need in-person review, and interacting with men via 2wT. One of the participants said the following:

On the texting side is that if there is no teamwork, if we can’t work together as a team, you will find yourself working alone, even on weekends, because now you’d be on the 2wT responding, doing things, and then there is no rest for you.

P4

#### Lack of Clarity on the Distinction Between 2wT Routine and Research Duties

HCWs expressed some confusion as to what tasks were part of the 2wT intervention and regarding the requirements for RCT-specific monitoring, leading to a lack of initial buy-in and confusion about 2wT follow-up workload. Research-specific tasks, including the duplication of patient reports in both routine paper (NDoH) and electronic research reporting (eg, 2wT and Google Doc [Google LLC]), added considerably to the staff’s workload. Remarking on the increased documentation, one of the HCWs lamented, *“*it’s really this recording of the same thing over and over and over in different places, you know, it’s that intake form, then it’s medic mobile [2wT system], then it’s the Google Doc, it can be quite time consuming, especially if you’re having a busy day at the clinic” (P3). Combined 2wT intervention and RCT tasks created an undue burden for routine HCWs who were supposed to perform VMMC and conduct follow-ups in addition to daily RCT monitoring:

When there are clients waiting...we had like 17 clients, we cut all those 17 clients in one day, it was a very busy day that we even finished around half past six. So, if we had to answer the phone, and enroll clients, update the Google Doc, it was it was impossible to happen. So, the challenge is the shortage of staff members, and maybe increase work for the staff member.

P2

Due to staff turnovers, HCWs received refresher training that enhanced their understanding of RCT versus routine activities. This added orientation was implemented to “make sure that and reassure people that two-way texting is part of your normal VMMC routine” (P4), hoping that *“*once people understand that it’s going to be part of routine care,...they will definitely buy into it” (P3).

#### Need to Address 24/7 Interaction Requests From 2wT

Men also responded via 2wT over weekends, after hours, and on public holidays, creating work for the clinician outside routine clinic hours. Although there is an automated 2wT response that indicates the clinic is closed and provides emergency numbers, one of the clinicians noted, *“*Usually, we do not work on weekends. So then, now...our clinician has to make sure that they check the system” (P1). Some clinicians “won’t take the phone over weekends or after hours because [they] see it as work and its overtime, and [they] want to be paid for it” (P3). However, another nurse noted that she feels personally responsible for the men, making herself available to interact with men who work nightshifts:

I always check are there any messages that might have come in late, because some guys work the night shift. So, they only respond when they go to work and they’ve got some time in between. Because during the day, they’re sleeping. So, you know, you won’t get a response from them. But only after hours, you get a response. So, I find that if I just check before I go to bed, whether it be 9 o’clock or whether it be 11 o’clock or midnight...just to make sure that there’s nothing.

P3

#### The 2wT System Excludes Some Clients

The 2wT system can potentially exclude clients based on their access to cell phones, literacy, disability, and other health dimensions. One of the HCWs stated, “[2wT] would be a problem for the ones that do not have access to cell phones” (P1). Another HCW suggested that illiteracy was also a limitation for clients by stating, “it would also become an issue for the ones who cannot read or write. So as much as it will help our VMMC clients, there will be a portion that might be left behind” (P1). Age could also be a limiting factor owing to potential unfamiliarity with technology and unwillingness to access health care via a phone, as one nurse stated, “...our generation, we are technologically aware. All those who are in their 40s and upwards? It was a little bit of a strange phenomenon, I would say. They don’t believe in such a way that I can just communicate with someone via phone, they still [prefer visits] physically” (P5). Finally, some clients require closer postoperative monitoring because of underlying comorbidities; in such cases, nurse-led clinical monitoring would be expedient:

For example, you have a diabetic. You can’t have them not coming in...and also immunocompromised people, you know, unless they are properly stable on the medication. You want to see them because obviously, things can go wrong quite quickly, if you don’t monitor them in person.

P3

#### Technology Challenges

Technological issues such as tablets with low memory or slow network connections affected enrollment, as VMMC clients required confirmation of an enrollment text or the response rate of HCWs to clients’ messages if the network was slow. In rural areas, this issue was felt more often because with 2wT, “you should be always making sure that you check the clients on the 2wT app. At times as we travel, there’s a network problem” (P4). Network issues could result in clients not being enrolled into 2wT “because we have to actually see the [2wT enrolment message] when we are doing enrolment before the client leaves the facility” (P1), or the client resorts to in-person visits*.* The lack of access to Wi-Fi at home was another limitation reported by a participant who worked after hours at home and reported challenges with “the data connection because sometimes when you are home, it’s not easy” (P2).

## Discussion

### Principal Findings

The 2wT system was considered usable, acceptable, and advantageous by all the HCWs in this formative, qualitative study in South Africa, providing benefits such as cost and time savings, ensuring quality patient care, and improving access to postoperative clinical support. These findings confirm previous results regarding the usability of 2wT for VMMC in Zimbabwe, where 2wT was also found to be usable, acceptable, and advantageous for HCWs to provide quality postoperative VMMC care [16-19,37]. In contrast to previous research that found low engagement in care among men [33-36], the HCWs in this study believed that 2wT enhanced their postoperative communication and connectedness with VMMC clients. They were confident that their clients were using the system to report AE concerns and found it easier than scheduling in-person follow-ups with the clients. However, despite the HCD process that optimized the 2wT intervention in the local context, 2wT was not without drawbacks for HCWs. There was a perception of increased work owing to the need to interact with clients via SMS text messaging while performing other routine tasks and the need to respond to men outside routine clinic hours. Network outages slowed enrollment and system response time in more rural areas, which reduced 2wT enthusiasm to some extent. Not all clients would benefit from 2wT, an issue that is also true for other digital innovations at scale. Overall, HCWs believed that the 2wT system is usable and acceptable, bringing benefits to both patients and providers. Several lessons learned warrant discussion and inform scale-up preparations.

First, using an HCD approach helped match the app adaptation to the local context. Familiarity with the use of mobile phone technologies such as WhatsApp (Meta Platforms, Inc) and SMS text message exchange improved HCWs’ ease of using and confidence with the system from study inception. Using this solid foundation, the HCD process ensured that the app features (reporting content and provider decision support), flows (clerk vs nurse 2wT activities), and alerts (tracing and referral closures) reflected the local VMMC program and service delivery in both rural and urban contexts. Consistent feedback sessions with HCWs as part of the HCD process resulted in a 2wT app that was user-friendly, intuitive, and supportive of clinical decision-making. Because of HCD, HCWs expressed that 2wT was easy to use, reducing anxiety and increasing the motivation to adopt 2wT for postoperative care. Moving forward, additional adaptations should also follow this cocreation process with HCWs, giving them a clear voice and opportunity to contribute to apps designed for their benefit.

Second, the TAM behavioral theory aided in the understanding of how and why 2wT’s usability and acceptability grew over time [30,32], informing the training model for scale-up. As expected from the TAM constructs, HCWs expressed growing confidence in using 2wT to support patients in their healing process, and 2wT provided HCWs with control over patient management. HCWs felt secure that they could use 2wT to help men with potential AEs identify and act on those events by referring them to care and reviewing them in person. HCWs noted that their self-efficacy increased by using 2wT to remotely conduct patient management and follow-up, triaging some patients to in-patient care but helping the vast majority to heal well at home. HCWs recognized that their confidence and capacity strengthened over time. For scale-up, HCWs suggested that training should be conducted in phases, with initial and refresher training on 2wT. Initial training could help gain HCW buy-in through a clear explanation of the rationale behind 2wT and the advantages of 2wT for both patients and providers, which increases motivation. Then, a series of practical, hands-on 2wT trainings with on-site follow-up mentoring could help ensure HCWs’ confidence and competence over time, solidifying gains.

Third, although the 2wT reduced workload in terms of in-person visits, 2wT does require HCWs to spend time on interacting with, and providing telehealth for, patients. Instead of routine postoperative reviews, providers regularly monitored the 2wT system, interacting with patients via SMS text messages and reporting any potential AEs within the 2wT system forms, which is a trade-off in terms of work activities but not a true reduction in work hours. Patients who responded after hours, or requested help during evenings and weekends, also conflicted with subjective institutional norms, where overtime work is compensated through overtime pay. In addition, service delivery patterns or seasonal scheduling, such as high face-to-face patient loads on certain days, limited HCWs time to respond to postoperative clients on the 2wT platform, delaying their and, in turn, their clients’ responses. These realities negatively impacted HCWs’ attitudes toward and use of 2wT, as evidenced by some HCWs’ refusal of or delay in replying to clients outside of clinic operating hours or during busy work hours. HCWs suggested that having additional personnel tasked specifically with 2wT duties or a rotation of nurses to handle off-hour or weekend calls would increase buy-in and reduce potential care delays.

Fourth, conducting an RCT in a routine public health care setting and relying predominantly on routine HCWs has clear advantages in terms of understanding real-world opportunities, working within existing constraints, and gaining insights into successful sustainability and scale-up. A research coordinator was deployed at each site for study-related activities (recruitment, enrollment, consent, and randomization), whereas routine VMMC teams were expected to complete all other routine VMMC-related tasks and NDoH forms for study participants as part of their normal duties without additional compensation. However, post hoc HCD sessions with routine HCWs and the RCT research team illuminated core confusion in research (RCT specific) versus routine (scale-up) activities, muddling workload perceptions. For example, on top of routine work, existing VMMC staff members were tasked with completing time-consuming daily study logs of enrolled patients’ clinical processes and completing both paper- (NDoH) and 2wT-based visit documentation, activities that took time but did not continue beyond the study. Additional sensitization would help clarify routine versus research tasks and potentially help HCWs distinguish temporary study-related documentation from the intervention itself. This could aid HCW buy-in and better set expectations for workload benefits. Continued conversations with relevant stakeholders, such as the NDoH and other MC implementation partners, on integrating 2wT into the current documentation systems would further lessen HCW workload and centralize important patient health information.

Finally, continued emphasis on the rigorous evaluation of digital innovations, especially in routine health service delivery settings, will strengthen the evidence base for mHealth optimization and expansion [8]. A comprehensive digital health monitoring and evaluation (M&E) process is critical for finding the right innovation for bridging an identified gap, with consistent and iterative assessment of progress to scale or discontinue activities [9]. For 2wT, the team placed emphasis on M&E from the beginning, incorporating close assessment from the design stage to help ensure local relevance, usability, and broad acceptance among HCWs. Through this process and through consistent feedback loops with HCWs, the study team aimed to bring the promise of 2wT workload benefits to bear, helping HCWs effectively manage patients without dramatically increasing their burden. Issues and concerns were closely monitored, documented, addressed whenever possible, and used for improvement. However, close M&E of the implementation failed to identify and clarify the confusion on key 2wT HCW tasks. More balanced attention to both the process (HCW activities) and outcomes (patient safety) could have rectified this issue earlier in the study implementation.

### Limitations

First, although 2wT’s usability among HCWs was high, the HCD process could not ensure that 2wT optimization addresses all the challenges of real-world implementation or incorporate all suggestions. The 2wT system is not a one-size-fits-all solution for weaknesses in VMMC programs but a focused digital health innovation aimed at addressing the dual concerns of quality and workload. If 2wT optimization supports most men who underwent VMMC in most locations to use 2wT successfully, HCWs and resources can be focused on attending to men with concerns through 2wT, conducting reviews for those who cannot be enrolled into 2wT, or addressing other gaps in VMMC service delivery. Second, patients’ experiences of 2wT were not included in this study but were explored in a parallel study. As with most qualitative studies, the small sample size reduces the diversity of perspective; additional KIIs in subsequent 2wT study phases will expand insights into 2wT scale-up. Furthermore, HCW participants’ experiences with the 2wT system were confounded with the experience of undertaking an RCT with its associated paperwork. Moreover, likely because of severe HCW scarcity and low resources, KIIs noted challenges with teamwork and workload distribution at the site level. Although beyond the scope of this paper, additional understanding of and amelioration of these work site constraints could improve the success of this or other innovations. Finally, although community health toolkit–based apps are optimized for low-connectivity and low-power settings, sporadic network issues that slowed or stymied client or provider communication lessened confidence in the system. As infrastructure improves, it is hoped that these challenges will reduce over time.

### Conclusions

HCWs involved in the 2wT post-VMMC follow-up approach found the system to be both usable and acceptable but also noted several improvements to bring 2wT to scale. Several suggestions were implemented immediately: (1) 2wT patient counseling was enhanced to better illustrate expected patient responses, reinforce AE warning signs, and remind patients that 2wT is not for emergencies; (2) educational messages were integrated with daily response messages to remind men about normal wound healing progress; and (3) cheat sheets were provided to 2wT nurses to guide daily task completion. Other suggested changes are more complex and require a more systematic or system-wide approach. HCWs noted that a rotation system, shift in work hours, or compensation for overtime was needed to interact with men using 2wT during nights, weekends, or holidays. Although these challenges are not easily addressed, additional HCW sensitization to promote the workload trade-off of 2wT interaction versus time-consuming, largely unnecessary physical postoperative visits could help facilitate HCW buy-in for the 2wT approach. Additional 2wT app modifications could also improve the intervention in the future. First, as HCWs principally use 2wT on smartphones or tablets to communicate with patients, a system-generated audible 2wT message notification, as is common with SMS text messaging or WhatsApp, could help provide an audible nudge to improve timely responses. Second, additional logic flows or machine learning could automate initial responses to common wound care concerns (eg, suggesting taking the provided tablets for pain and reminding men to elevate the penis to reduce swelling), relieving the workload of responding to expected, low-risk questions or requests for reassurance. Overall, HCWs enjoyed the process of providing feedback and insights. Attention to both HCW cocreation and the incorporation of HCWs’ suggestions would ensure continued HCW buy-in and support for 2wT scale-up.

## Abbreviations

2wT: two-way texting
AE: adverse event
CHAPS: Centre for HIV-AIDS Prevention Studies
HCD: human-centered design
HCW: health care worker
KII: key informant interview
LMIC: low- and middle-income country
M&E: monitoring and evaluation
MC: male circumcision
mHealth: mobile health
NDoH: National Department of Health
RCT: randomized controlled trial
SoC: standard of care
SSA: sub-Saharan Africa
TAM: technology acceptance model
VMMC: voluntary medical male circumcision

## Data Availability

The data sets generated during or analyzed during this study are available from the corresponding author upon reasonable request.
